# Advances in understanding the pathogenesis of epilepsy: Unraveling the molecular mechanisms

**DOI:** 10.1002/hsr2.1896

**Published:** 2024-02-14

**Authors:** Sanobar Shariff, Halah A. Nouh, Samuel Inshutiyimana, Charbel Kachouh, Maya M. Abdelwahab, Abubakar Nazir, Magda Wojtara, Olivier Uwishema

**Affiliations:** ^1^ Oli Health Magazine Organization, Research and Education Kigali Rwanda; ^2^ Department of Medicine Yerevan State Medical University Yerevan Armenia; ^3^ Department of Medicine Lebanese University Beirut Lebanon; ^4^ Department of Medicine United States International University‐Africa Nairobi Kenya; ^5^ Department of Medicine Saint‐Joseph University Beirut Lebanon; ^6^ Faculty of Medicine Helwan University Cairo Egypt; ^7^ Department of Medicine King Edward Medical University Lahore Pakistan; ^8^ Department of Medicine University of Michigan Medical School Ann Arbor Michigan USA; ^9^ Department of Medicine Clinton Global Initiative University New York New York USA; ^10^ Faculty of Medicine Karadeniz Technical University Trabzon Turkey

**Keywords:** antiepileptic pharmacotherapy, epilepsy, epileptogenesis, gene therapy, precision medicine

## Abstract

**Introduction:**

Epilepsy is characterized by having two or more unprovoked seizures. Understanding the pathogenesis of epilepsy, requires deep investigation into the molecular mechanisms. This helps develop diagnostic techniques, treatments, and pharmacotherapy. It also enhances precision medicine and individualized treatment processes. This article reviews all the molecular mechanisms predisposing to epileptogenesis, presents the current diagnostic techniques and drug therapy, and suggests future perspectives in treating Epilepsy in a more comprehensive and holistic approach.

**Methodology:**

Four authors searched keywords concerning epilepsy at a molecular level, Epilepsy diagnostic techniques and technologies, and antiepileptic drug therapy and precision medicine. Separate search strategies were conducted for each concern and retrieved articles were reviewed for relevant results.

**Results:**

The traditional diagnostic techniques for Epilepsy and its pathogenesis are insufficient in highlighting dynamic brain changes. For this, emerging technologies including genetic sequencing and profiling, and functional neuroimaging techniques are prevailing. Concerning treatment, the current approach focuses on managing symptoms and stopping seizures using antiseizure medications. However, their usage is limited by developing resistance to such drugs. Some therapies show promise, although most antiseizure drugs do not prevent epilepsy.

**Discussion:**

Understanding epileptogenesis at a molecular and genetic level aids in developing new antiepileptic pharmacotherapy. The aim is to develop therapies that could prevent seizures or modify disease course, decreasing the severity and avoiding drug resistance. Gene therapy and precision medicine are promising but applications are limited due to the heterogeneity in studying the Epileptic brain, dynamically. The dynamic investigation of the epileptic brain with its comorbidities works hand‐in‐hand with precision medicine, in developing personalized treatment plans.

## 1 INTRODUCTION

The medical condition of epilepsy is characterized by two or more seizure episodes and uncontrolled and unprovoked firing of the brain neurons. This chronic disease is noncommunicable, and it affects an estimated number of 50 million people, all ages included, globally.^1^, ^2^ Seizures result from inadequate or excessive firing of the neurons, and this culminates in the inability of the brain to coordinate the rest of the human body. It affects processes such as limb as well as bowel movements and may take a varying frequency from several seizures per day to less than one per year. Epilepsy can affect one region of the body (focal epilepsy) or the whole body (generalized epilepsy) depending on the location of the affected neurons in the brain.

Epilepsy occurs because of genetic susceptibility, neural development, cerebrovascular factors, and other acquired factors that irritate neural tissues. Additionally, it can occur due to neoplasm, metabolic, or neurodegenerative disorders, especially in old people. All the mentioned factors have the potential to cause damage to the brain and consecutively alter the neuronal circuitry.^1^ Thus, epilepsy accounts for sudden unexplained deaths of people each year. Worldwide, approximately 5 million people suffer from epilepsy. In high‐ and low‐income countries, the disease is projected to affect 49 out of every 100,000 and 139 per 100,000 people respectively. It has been observed that the rise in malaria and neurocysticercosis cases, accidents pertaining to road traffic, accessibility of preventive health programs, birth injuries, and available care are the culprits pertaining to epilepsy cases.^1^ Consequently, this begs a question toward an advanced understanding of the pathogenesis of epilepsy with a focus on reducing its burden on the development of humankind. The development of new antiepileptic medicines that treat epilepsy symptomatically, influence epilepsy's evolution, or prevent epileptogenesis will probably be the goal of an advanced understanding of epilepsy's pathogenesis.

This review sheds light on molecular biology particularly the genetic factors and mechanism of epileptogenesis to facilitate the practice of treating epileptic patients. Additionally, emerging technologies for investigating the pathogenesis of epilepsy are highlighted in this review.

### 1.1 Classification and types of epilepsy

To improve communication between patients and health professionals, the International League Against Epilepsy (ILAE) in 2017 set guidelines that incorporate comorbidities and etiological factors in the classification of epilepsy.^3^ Thus, three levels are followed in the classification of epilepsy. The first level entails the identification of the seizure type which informs the type of epilepsy in the second level. The last level focuses on finding out the presence of epileptic syndrome wherein a patient is affected by multiple epilepsy types.^4^ The main types of epilepsy are four based on the seizure type that is present. These types include focal, generalized, combined focal generalized, and unknown type. Furthermore, it is crucial to note that patients with repeated provoked seizures or single seizures are not epileptic.^3^ The type of epilepsy is unknown wherein the clinicians cannot categorize seizures as either generalized or focal. When a patient is diagnosed with both focal and generalized seizures, the epilepsy type called combined generalized and focal epilepsy is confirmed, and this condition is commonly found in cases of severe epilepsies associated with children or infants. Generalized epilepsy encompasses a wide range of seizure forms, and a patient may present any sort of generalized seizure, nonmotor, or motor. Besides, focal epilepsy is a spectrum condition characterized by seizures that are restricted to a single hemisphere.^4^

### 1.2 Genetic factors in epilepsy

The etiology of epilepsy can be unknown, immune, metabolic, infectious, structural, or genetic in nature. Several epileptogenic de novo mutations have been classified as inborn, but it is important to note that genetic mutations are not always inherited. Any cause of mutations in the genetic makeup of brain neurons will eventually impair the excitability of such neurons in the brain hence genetic etiology.^3^ Genetic forms of epilepsy have been linked to the role of specific ion channels particularly voltage‐gated sodium channels by epileptogenesis and epilepsy molecular studies because both GABAergic and glutamatergic cell types express several types of sodium channels.^5^ Generalized epilepsy associated with febrile seizures plus (GEFS+) occurs when voltage‐gated sodium ion channels (NaV1.1) undergo missense mutations. Furthermore, familial febrile seizures are a result of slight loss of function mutations in NaV1.1 channels, which take part in vaccination‐related febrile convulsions. Moreover, calmodulin, a tiny protein that responds to calcium levels at the synapse and influences the voltage‐gated sodium‐channel gating process, regulates the protein expression of sodium channels. Numerous mutations in the calmodulin‐binding IQ domain result in epilepsy.^4^ The Dravet syndrome that occurs due to SCN_1_A mutations and the Benign Familial Neonatal Epilepsy which results from mutations of KCNQ_3_ or KCNQ_2_ have been identified as genetic epilepsies up to date^3^ acetylcholine (ACh), the primary autonomic nervous system stimulant, promotes signal transmission via cholinergic and nicotinic receptors. Recent studies suggest that nicotinic ACh receptor dysfunction, which is extensively expressed in hippocampus and cortical neurons, may have a key role in the pathophysiology of epilepsy.^6^

## 2 DISCUSSION

### 2.1 Molecular mechanisms of epileptogenesis

Molecular mechanisms of epileptogenesis are complex and not fully understood, but they are thought to involve an imbalance between excitatory and inhibitory signaling in the brain, abnormal synaptic plasticity network hyperstability, inflammation, and immune dysregulation. Therefore, the primary endpoint of this study is to analyze how these factors can lead to epilepsy.

#### 2.1.1 Excitatory and inhibitory neurotransmitter imbalance

Most seizures in humans are caused by chronic epilepsy rather than toxic exposure, and this situation requires the idea of an imbalance between inhibitory and excitatory conductance to be expanded.^7^ A seizure happens when there is a decrease in inhibitory signaling such as gamma‐aminobutyric acid (GABA) or an increase in excitatory signaling such as Glutamate.^7^ Mutations in genes encoding for synapsins have been shown in clinical studies to be associated with epilepsy and their deletion may cause excitatory/inhibitory imbalance which can eventually lead to seizures.^7^ Mutations in the stargazin gene, a member of the AMPA receptor regulatory protein (TARP) family, are one example, and mutations in the LGI1/ADAM22 genes, which connect stargazin to the postsynaptic density, may both cause epilepsy^7^(Table 1).

**Table 1:** Different etiologies for epileptogenesis.

Factor	Description	References
Etiology of epileptic seizures	Chronic epilepsy, not toxic exposure.	[7]
The imbalance between inhibitory and excitatory conductance	Decreased inhibitory signaling (GABA) or increased excitatory signaling (Glutamate).	[7]
Synapsins	Mutations in synapsins' genes are associated with epilepsy.	[7]
Relation between synapsins and epileptogenesis	Deletion of synapsins may cause excitatory and inhibitory neurotransmitter imbalance.	[7]

#### 2.1.2 Abnormal synaptic plasticity and network hyperexcitability

Other mechanisms may be involved in epilepsy. First, mutations in genes encode ion channels. Ions can cross cell membranes thanks to ion channels (i.e., proteins). Ion channel genes can be altered by mutations, pathogens, or antibodies. Suggested authors' studies showed that increased amounts of antibodies participate in infiltrating CD8+T cells which hold cytotoxic granules that may lead the neuron's ion channel to block, therefore changing the way ions enter and leave neurons.^7^, ^8^ Second, synaptic plasticity is the ability of synapses to change in strength over time. Changes in the strength of synapses that occur over seconds could be a potential mechanism of seizure generation, which can also be associated with inhibitory synapses.^7^, ^8^ Macrophage inflammatory proteins (MIP) and interleukin‐6 (IL‐6) are proteins secreted by astrocytes and microglia that can facilitate hyperexcitability.^8^ The role of several immunological modulators in neural plasticity and synaptic transmission has been demonstrated.^8^ The suggested dynamic modulation of excitation and inhibition during stimulation at gamma and beta frequencies in the hippocampal region might generate an immediate increase in synapse strength (Table 2).

**Table 2:** Abnormal synaptic plasticity and network hyperexcitability involvement in epileptogenesis.

Factor	Description	References
Mutations in ion channel genes	Ion channels allow ions to pass through cell membranes. Mutations, infections, and antibodies can all affect ion channel genes.	[7, 8]
Antibodies and ions channels	Increased antibodies and CD8+ T cells can block ion channels in neurons, altering ion flow.	[7, 8]
Synaptic plasticity and network hyperexcitability	Synapses can change strength instantaneously and could be a potential mechanism of seizure generation.	[7, 8]
Inhibitory synapse	Synaptic pliancy can likewise be related to the inhibitory neural connection.	[7, 8]
Immune modulators (cytokines)	Macrophage inflammatory proteins and interleukin‐6 have shown their involvement in epileptogenesis.	[7, 8]
Gamma and beta frequencies	Dynamic changes in stimulation and constraint during sensation at γ and β frequencies in the hippocampus region can result in a rapid increase in neural connection strength.	[9, 10]

#### 2.1.3 Role of inflammation and immune dysregulation

Inflammation and immune deregulation can also play a role in triggering an epileptic seizure. Inflammatory cells release molecules that can alter neuronal signaling, which can lead to seizures. During the epileptic phase, increased levels of IL‐6 and tumor necrosis factor (TNF) were observed when examining synaptic protein expression, brain inflammation, and adult hippocampus neurogenesis in mice lacking synapsin 2.^9^ Currently, central nervous system (CNS) inflammation caused by blood–brain barrier (BBB) leakage is associated with the induction and progression of epilepsy.^8^ Large deposits of extravasated immunoglobulin G (IgG) are found in seizure‐generation areas of the brain, defining the BBB disruption.

Furthermore, the immune system is thought to have a role in the development of epilepsy. The presence of IgGs in the brain in seizure disorders is evidence of auto‐immune involvement in the pathogenesis.^10^ Encephalitis can also trigger cell mortality; especially status epilepticus‐induced neuronal cyclooxygenase 2 overexpression during epileptogenesis has been shown to have a substantial role in neuronal cell death^11^ (Table 3).

**Table 3:** The factors related to inflammation and immune dysregulation that can promote epileptic seizures.

Factor	Description	References
Inflammation	Inflammation can alter neural signaling.	[9]
Interleukin‐6	Increased values may be associated with seizures.	[9]
Tumor necrosis factor	Increased values may be associated with seizures.	[9]
Blood–brain barrier	BBB disruption releases large amounts of IgG.	[8]
Immunoglobulin G	If present in the brain, the etiology of seizures may be auto‐immune.	[9]
Encephalitis	Can promote cell loss.	[11]
Cyclooxygenase 2 (COX‐2)	Neuronal cell death may be caused by COX‐2 upregulation caused by status epilepticus.	[12]

### 2.2 Emerging technologies for investigating epilepsy pathogenesis

Physical examination, EEG analysis, CT scan, and MRI are considered current methods for investigating epilepsy pathogenesis. These methods are limited by their low resolution and their inability to capture the dynamic changes that occur in the brain during an epileptic seizure.^13^ Besides lacking accuracy in diagnosis and investigation, patients may be exposed to hazardous radiation and may present allergies to certain contrast materials.^13^ Emerging technologies such as advances in genetic sequencing and expression profiling, neuroimaging techniques for studying the brain's activity and connectivity, and in vivo studies on various animal models and how they contribute to understanding epilepsy mechanisms are being used to help in investigating epilepsy pathogenesis (Table 4).

**Table 4:** The current imaging methods and their limitations in epilepsy diagnosis.

Imaging method	Description	Limitations	References
Physical examination	Assessment of the patient's body for indications of epilepsy, like unusual developments or conduct.	Not very specific to epilepsy.	[13]
EEG analysis	Recording of the electrical movement of the cerebrum.	Epilepsy's etiology cannot be identified by EEG, but abnormal electrical activity in the brain can be.	[13]
CT scan	Brain imaging using X‐rays.	Epilepsy's cause cannot be identified by EEG, but abnormal electrical activity in the brain can be.	[13]
MRI	Brain imaging using a magnetic field and radio waves.	MRI can identify mind anomalies and seizure movement but with lower resolution.	[13]

#### 2.2.1 Advances in genetic sequencing and gene expression profiling

In almost half of people, there is thought to be an underlying genetic propensity for epilepsy.^14^ Finding the genes that contribute to monogenic types of epilepsy may have advanced significantly over the past 15 years because of developments in genetic sequencing and gene expression profiling,^14^ Next‐generation sequencing (NGS), which is a fast, cost‐efficient method, consists of sequencing the protein‐coding regions and, therefore, sequencing the exomes.^14^ Exomes sequencing and gene panels are a comprehensive and nondiscriminatory approach to identifying genetic variants that may be associated with pathogenesis.^14^, ^15^ Epileptic encephalopathy (EE), the most severe form of epilepsy, was previously thought to be a sporadic or acquired disorder. Recent exome sequencing studies highlight the importance of novel mutations in EE. De novo mutations in the SCN1A gene and the DNM1 gene may lead to Dravet syndrome and EE respectively^15^, ^16^, ^17^ (Table 5).

**Table 5:** Advances in genetics for epilepsy study.

Factor	Description	References
Genetic sequencing	Gene sequencing aids in the discovery of genes associated with monogenic forms of epilepsy.	[14]
Next‐generation sequencing	Fast and cost‐efficient method when compared to normal genetic sequencing.	[14]
Genes panels and exomes sequencing	A comprehensive strategy for locating pathogenic genetic variants.	[14, 15]

#### 2.2.2 Neuroimaging techniques for studying brain activity and connectivity

Traditional methods used to study anatomical connectivity in animals have relied on histological techniques, which have provided valuable insights into neural circuit connectivity with little direct information in humans. Neuroimaging techniques for studying the brain's activity and connectivity may also help in the diagnosis and investigations of epilepsy pathogenesis. Measuring structural brain connectivity and collecting functional neuroimaging data is common among the different techniques.^18^ The most popular method for assessing synchronization is to compute the correlation between brain activity time series taken from the most frequent area of concern (i.e., temporal lobe region).^18^ The use of ultrahigh‐field imaging and postprocessing approaches can help identify lesions, particularly localized cortical dysplasia and hippocampal sclerosis. In finding focal abnormalities in MRI‐negative patients, statistical analysis of positron emission tomography and single photon emission computed tomography outperforms qualitative analysis alone.

These techniques have also been utilized to investigate epileptogenesis and medication resistance mechanisms^19^, ^20^ (Table 6).

**Table 6:** Advanced neuroimaging techniques for investigating epileptogenesis.

Neuroimaging techniques	Description	References
Structural brain connectivity	Can be quantified utilizing neuroimaging strategies.	[18]
Functional neuroimaging data (i.e., fMRI)	Can be obtained through neuroimaging methods.	[18]
Quantifying synchronization	Given registering connection among the cerebrum actuation time series recorded from the most continuous area of interest.	[18]
Ultrahigh‐field imaging	Can work on identifying sores, particularly central cortical dysplasia, and hippocampal sclerosis.	[18]
Positron emission tomography/single photon emission computed tomography statistical analysis	In MRI‐negative individuals, qualitative analysis is superior to quantitative analysis in recognizing central abnormalities.	[19, 20]

#### 2.2.3 Animal models and their contributions to understanding epilepsy mechanisms

In vivo, studies using various animal models can contribute to understanding epilepsy mechanisms despite having experimental limitations. Mimicking the natural history of symptomatic focal epilepsy remains quite difficult, animal models should be classified into three separate categories: genetic, chemical, and traumatic brain injury models.^21^ In selecting animal model categories, the age factor remains essential. Elderly people may often take more medications and have various comorbid conditions, which can give false‐positive results thus conveying a greater predisposition for the first seizure^22^ (Table 7).

**Table 7:** The contribution of animal models to epileptic seizure mechanisms.

Animal models category	Description	References
Genetic models	These models are created by introducing mutations into the genes of animals that are known to be involved in epilepsy.	[22]
Chemical models	These models are made by infusing chemicals into the brains of animals that are known to trigger seizures.	[22]
Traumatic brain injury models	These models are being selected to attempt to trigger epileptic seizures by causing brain wounds.	[22]
Age factor	Remains essential in selecting animal models as elderly animals may have different seizure thresholds than younger animals.	[23]

### 2.3 Molecular targets for antiepileptic drug development

#### 2.3.1 Overview of current antiepileptic drugs and their limitations

The current treatment for epilepsy focuses on managing symptoms and stopping seizures using antiseizure medications, which act through various mechanisms, such as blocking voltage‐gated calcium and sodium channels, enhancing the inhibition of GABAergic, and reducing transmission of excessive excitatory amino acid.^24^ Recent studies have identified alternative treatments, such as the stimulation of the vagus nerve and a ketogenic diet. However, around 30% of patients still have drug‐resistant epilepsy.^25^ Developing new methods for epilepsy pharmacotherapy is challenging due to various factors. Most antiseizure medications have been identified through screening tests of animals or by modifying the existing drugs' chemical structures. Only a few have been designed upon their neurochemical mechanism of action.^26^ However, progress in multi‐omics methods and new drug discovery strategies, such as fragment‐based drug discovery and virtual and high‐content screening, may expedite the discovery of novel molecules that demonstrate promising effectiveness in preventing seizures. To increase the number of effectively treated patients, it may be necessary to select new targets for seizure suppression or focus on disease modification or prevention.^22^

#### 2.3.2 Potential molecular targets for novel drug development

Targeting the biological processes involved in developing epilepsy, known as epileptogenesis, is a promising strategy for preventing epilepsy.^27^ Despite the existing knowledge about epileptogenesis, there have been limited efforts to target specific molecular mechanisms for preventing or altering epilepsy development or symptoms, and no therapeutic intervention has been successfully developed thus far. Therapies could prevent seizures altogether or modify the course of the disease to decrease the severity and drug resistance. Antiepileptogenic strategies have focused on preventing neuronal cell death, inhibiting neuroinflammation, blocking glutamate signaling, targeting neurotrophin pathways, and modifying epigenetic processes.^26^ Some show promise, like brivaracetam, which targets synaptic vesicle protein 2A, though most antiseizure drugs do not prevent epilepsy.^27^ Mechanisms likely differ based on injury type and location but involve astrocyte activation, mTOR signaling, inflammation, glial activation, and cell communication (Table 1). Analyses of human and animal tissues and transcriptomic and proteomic analyses have revealed molecular networks and noncoding RNAs involved.^28^ However, the complexity of interactions makes antiepileptogenic drug development challenging. Beyond drugs, gene and cell therapies may provide targeted disease modification. Combination strategies targeting multiple mechanisms may ultimately be needed. The most promising targets regulate neuroplasticity, neuroinflammation, and excitatory/inhibitory signaling. New techniques like optogenetics and connectomics could help rewire pathological circuits. Translational research in human and animal tissues is critical to developing antiepileptogenic and true antiepileptic therapies.^29^

#### 2.3.3 Advances in precision medicine and personalized treatment approaches

Recent advances in genetic testing have revealed a genetic etiology is accounting for over half of the cases. Inherited forms of epilepsy are predominantly attributed to single gene defects.^30^ Precision medicine aims to develop treatments tailored to a patient's specific pathophysiology, anticipating the efficacy of specific prevention and treatment approaches for particular groups of individuals. Nevertheless, the application of precision medicine in routine healthcare remains limited.^31^ Epilepsy offers a chance to tailor treatments to individuals, as multiple genes contribute to its etiology. It has shown promise in certain epilepsy syndromes, such as peroxide‐dependent epilepsy, Dravet syndrome, and glucose transporter 1 deficiency, but it has mainly focused on seizure control. Nonetheless, this approach remains challenging due to the heterogeneity of epilepsy and its underlying causes.^32^

### 2.4 Role of neuroinflammation in epilepsy

#### 2.4.1 Immune‐mediated mechanisms in epileptogenesis

While inflammation in the brain has been shown to contribute to epileptogenesis, there are also immune responses that are protective and stimulate neuronal repair. Therefore, it is essential to investigate whether immune responses observed during the onset and progression of epilepsy are exclusively harmful to the brain cells' survival or if they serve neuroprotective purposes as well.^33^ The current understanding is that both brain‐resident cells of innate immune responses and peripherally derived innate and adaptive immune cells contribute to immune‐mediated epileptogenesis. Various triggers, such as febrile seizures, stroke, infection, or trauma, can initiate an inflammatory cascade that releases pro‐inflammatory cytokines, such as IL‐1β and TNF‐α, and danger signals, such as HMGB1. These factors stimulate neuron pathways, resulting in dysregulated ion channels, neuronal hyperexcitability, and a reduced seizure threshold. Additionally, pro‐inflammatory cytokines can stimulate the persistent release of excitatory neurotransmitters, inhibit their uptake, and restrict the recycling of GABA receptors.^34^ Also, COX‐2 and prostaglandin can be involved in this process, mobilizing intracellular calcium storage and increasing cAMP production resulting in the remodeling of the neuronal network.^35^ CNS inflammation can also lead to BBB leakage that introduces blood components into the brain and allows leukocyte infiltration. Activated peripheral immune cells can generate free radicals and release additional cytokines, chemokines, nitric oxide, and cytotoxic enzymes, further contributing to epileptogenesis.^36^

#### 2.4.2 Inflammatory markers and their association with epilepsy

Neurons and Glial cells mainly produce cytokines throughout brain inflammation. Sinha et al. suggested that cytokine production in the brain is triggered by seizure activity, which can lead to nerve cell damage and increased excitability. ^36^ Studies have shown that seizures cause an increase in pro‐inflammatory cytokines, including IL‐1β, IL‐2, IL‐4, IL‐6, IFN‐γ, and TNF‐α.^37^, ^38^ Elevated levels of IL‐1β, IL‐6, IL‐9, and TNF‐α have been linked to the subsequent onset of epilepsy in children who experienced acute seizures.^38^ Refractory epilepsy patients had remarkably increased levels of IL‐6 in their serum in comparison to healthy people. Also, IL‐6 level was more elevated in patients on polytherapy.^39^ Cytokine CCL2 is involved in neurodegenerative diseases and is upregulated in patients with intractable epilepsy.^40^ The CXCR2 receptor showed increased expression in temporal lobe epilepsy (TLE) patients, and using a CXCR2‐selective antagonist could inhibit its upregulation.^41^ The chemokine CXCL13 and its receptor CXCR5 were upregulated in epilepsy patients' brain tissue.^42^ TNF‐α is associated with epilepsy pathogenesis and correlated with seizure recurrence. It acts through the TNFR1 receptor, which increases glutamate transmission.^43^

#### 2.4.3 Potential therapeutic interventions targeting neuroinflammation

The current evidence supporting the therapeutic benefits of counteracting inflammation in epilepsy is limited. However, increasing evidence suggests that inflammation may contribute to seizures and epileptogenesis. As a result, anti‐inflammatory agents, particularly interleukin converting enzyme (ICE)/caspase‐1 inhibitors, are considered potential candidates for developing novel antiepileptic drugs (AEDs).^44^ Inhibition of ICE/caspase‐1 reduces IL‐1β release, decreases acute seizure activity, and restricts the generalization of seizures in preclinical models. These effects are associated with the downregulation of IL‐1β in astrocytes in the hippocampus.^45^ VX‐765, a good oral bioavailability prodrug, its active metabolite can cross the BBB. It has shown promise in preclinical models and it is used recently in a phase 2a clinical trial conducted on drug‐resistant partial epilepsy patients. Initial findings indicate that the treatment is safe and well‐tolerated. Later, a phase 2b trial is scheduled to assess its effectiveness and long‐term safety.^46^ These findings suggest that anti‐inflammatory strategies may be a viable treatment option for chronic epilepsy.^47^

### 2.5 Epilepsy and comorbidities

#### 2.5.1 Cognitive and psychiatric comorbidities associated with epilepsy

The epileptic brain is identified with its hyperexcitable nature of the neural circuity. However, recent literature is studying epilepsy in a more functional framework, shifting from investigating a strict molecular activity to highlighting the dynamical role of neural circuity between various brain regions.^48^, ^49^ Thus, explaining the comorbid cognitive and psychiatric symptoms associated with epilepsy.

Cognitive and affective comorbidities are prevalent in children and adults with epilepsy,^50^, ^51^, ^52^, ^53^ with TLE being the most common form^54^, ^55^, ^56^, ^57^ in adults, due to the temporal lobe's essential role in emotions and memory. Frontal lobe epilepsy (FLE) has been identified as well.^58^ In addition, pediatric epilepsy is categorized separately with cognitive, behavioral, and affective comorbidities, as well as, distinct sleep disturbance phenotypes.^59^, ^60^ Pediatric comorbidities are often a consequence of neurodevelopmental disorders, mainly involving autism spectrum disorder (ASD) and attention‐deficit hyperactivity disorder.^61^, ^62^ The clinical symptomatology of various comorbidities is summarized in Table 1.

Psychiatric comorbidities, either escort the epileptic clinical symptomatology or even precede it, with anxiety symptoms being more common at the time of diagnosis.^63^ Besides, cognitive disturbances may also precede the onset. Previous brain damage, longer seizure duration, interictal epileptic discharges, having a history of depression or anxiety, and surgical and medical interventions may be factors that elucidate such cognitive impairments,^64^ helping in predicting them^65^ (Table 8).

**Table 8:** The clinical symptomatology of cognitive, affective, and psychiatric comorbidities associated with epilepsy.

Epilepsy	Cognitive comorbidities	Affective and psychiatric comorbidities	Sleep disturbances
Temporal lobe epilepsy	‐ Posterior language disturbances^56^ ‐ Mild cognitive impairment including memory and language disturbances^53^	‐ Somatization^51^ ‐ Obsessive‐compulsive^51^ ‐ Interpersonal sensitivity^51^ ‐ Depression^51^ ‐ Anxiety^51^ ‐ Paranoid ideation^51^ ‐ Psychoticism^51^	
Frontal lobe epilepsy	‐ Task‐related attention deficit^56^ ‐ Task‐related executive Dysfunctions^56^		
Unidentified		Ictal panic^63^	
Pediatric	Decreased cognitive abilities^58^	‐ Behavioral dysfunction^57^ ‐ Depression^57^ ‐ Interictal dysphoria^57^	‐ Minimal^58^ ‐ Moderate^58^ ‐ Severe^58^ (The older the age of onset, the minimal the disturbance Oppositely, the younger the age of onset, the more severe the disturbance)^58^
	‐ Autism spectrum disorder^59^ ‐ Attention‐deficit hyperactivity disorder^59^ ▪ Response inhibition ▪ Aggressive behavior		

#### 2.5.2 Shared molecular mechanisms between epilepsy and other disorders

The etiologic categories of epilepsy vary between structural, genetic, infectious, metabolic, immune, and unknown.^59^ Emerging evidence suggests a significant association of the same gene mutations between epilepsy and neurodevelopmental disorders. However, this association is not a cause‐effect relationship, but rather it is a single continuum with various overlapping.^59^ To add, the frequency of epilepsy in children with ASD outstands that in typically developing children,^64^ with noted epileptiform abnormalities shown on encephalograms, even in the absence of clinical seizures.^65^ It is hypothesized, by small sample studies, that the alteration or the deviation of brain circuitry in ASD children might be normalized by the interictal epileptiform discharges,^65^, ^66^ leading to epilepsy. However, the effect of such discharges might surpass the tolerance level, elucidating autistic symptoms.^65^ Besides, the adenosine system provides balance for neuronal excitability and modulating seizures. An alteration in endogenous adenosine or adenosine receptors might be an underlying cause of epilepsy, along with other comorbidities including cardiovascular, cognitive, and sleep disorders.^66^

#### 2.5.3 Implications for personalized treatment strategies

In the meantime, drug development has not shown any promising effect in restraining epileptic seizures.^48^ In addition, antiseizure medications (ASMs) have various side effects on the brain and its hyperexcitability regulation, with an absence of epilepsy progression modification, and a significant occurrence of seizures despite the use of ASMs.^66^ This is explained by disregarding the neuropsychiatric comorbidities associated with epilepsy. For this, current efforts should be directed toward a holistic approach to treating epilepsy. Personalized neuropsychological assessment and comorbidities identification and treating, accordingly, is suggested. Lastly, in the pharmacotherapy field, studies should consider the side effects, as well as the overlap between epilepsy and associated comorbidities to better aid in providing more tailored interventions.^59^

## 3 CONCLUSION

### 3.1 Key findings

Traditional investigatory approaches for epileptogenesis are deficient in catching dynamic brain changes. For this, emerging technologies including genetic sequencing and profiling, and functional neuroimaging techniques are prevailing.

In terms of management, the current approach focuses on managing symptoms and stopping seizures using antiseizure medications, this use is limited by resistance toward such drugs. Preventing epilepsy includes targeting the biological processes involved. Some therapies show promise, though most antiseizure drugs do not prevent epilepsy.

### 3.2 Areas for future research and advancements

The goal of understanding pathogenesis is to develop new antiepileptic pharmacotherapy. This is challenging due to the limited number of medicines rationally developed based on their mechanism of action. Therapies that could prevent seizures or modify the disease course, decreasing the severity and drug resistance are what is sought. To enhance effectiveness, it is important to find new targets for seizure suppression.

Gene therapy and precision medicine are promising in predicting targeted treatment options. However, this approach is limited due to heterogeneity between various groups. The dynamic investigation of the epileptic brain with its comorbidities is a continuum of precision medicine, although clinical applications are still limited, it shows a promising role in developing personalized treatment plans.

### 3.3 Potential impact on epilepsy management and patient outcomes

This review aims to investigate the biological predisposition behind epileptogenesis. It discusses new technologies at the diagnostic level and suggests treatment options and advancements in the effort to put precision medicine and personalized treatment strategies into action. Therefore, assisting the future development of treatment and enhancing practice with epileptic patients.

## AUTHOR CONTRIBUTIONS

**Sanobar Shariff**: Supervision; writing—original draft; writing—review and editing. **Halah A. Nouh**: Writing—original draft; writing—review and editing. **Samuel Inshutiyimana**: Writing—original draft; writing—review and editing. **Charbel Kachouh**: Writing—original draft; writing—review and editing. **Maya M. Abdelwahab**: Writing—original draft; writing—review and editing. **Abubakar Nazir**: Visualization; writing—original draft; writing—review and editing. **Magda Wojtara**: Writing—original draft; writing—review and editing. **Olivier Uwishema**: Supervision; visualization; writing—original draft; writing—review and editing.

## CONFLICTS OF INTEREST STATEMENT

The authors declare no conflict of interest.

## TRANSPARENCY STATEMENT

The lead author Abubakar Nazir affirms that this manuscript is an honest, accurate, and transparent account of the study being reported; that no important aspects of the study have been omitted; and that any discrepancies from the study as planned (and, if relevant, registered) have been explained.

## Data Availability

The authors have nothing to report.
